# *Mycobacterium ulcerans* Disease (Buruli Ulcer) in Rural Hospital, Southern Benin, 1997–2001

**DOI:** 10.3201/eid1008.030886

**Published:** 2004-08

**Authors:** Martine Debacker, Julia Aguiar, Christian Steunou, Claude Zinsou, Wayne M. Meyers, Augustin Guédénon, Janet T. Scott, Michèle Dramaix, Françoise Portaels

**Affiliations:** * Institute of Tropical Medicine, Antwerp, Belgium;; † Centre Sanitaire et Nutritionnel Gbemoten, Zagnanado, Benin;; ‡ Armed Forces Institute of Pathology, Washington, DC, USA;; § Ministère de la Santé, Cotonou, Benin;; ¶ Ecole de Santé Publique, Brussels, Belgium

**Keywords:** Buruli ulcer, epidemiology, Mycobacterium ulcerans, skin ulcers, Benin

## Abstract

Data from 1,700 patients living in southern Benin were collected at the Centre Sanitaire et Nutritionnel Gbemoten, Zagnanado, Benin, from 1997 through 2001. In the Zou region in 1999, Buruli ulcer (BU) had a higher detection rate (21.5/100,000) than leprosy (13.4/100,000) and tuberculosis (20.0/100,000). More than 13% of the patients had osteomyelitis. Delay in seeking treatment declined from 4 months in 1989 to 1 month in 2001, and median hospitalization time decreased from 9 months in 1989 to 1 month in 2001. This reduction is attributed, in part, to implementing an international cooperation program, creating a national BU program, and making advances in patient care.

## Abstract 2

Hospital data show that Buruli ulcer is highly endemic in southern Benin.

Buruli ulcer (BU), caused by *Mycobacterium ulcerans*, is the third most common mycobacterial disease in humans after tuberculosis and leprosy (1). Endemic foci exist in tropical Africa, the Americas, Australia, and Asia (1–3). In 1997, the World Health Organization recognized BU as an emerging public health problem. Prevalences have increased during the last few years, especially in West Africa (4–7).

*M. ulcerans* is an environmental mycobacterium associated with wetlands, especially slow-flowing or stagnant water (8–10). Infection is often related to specific trauma (11). Aquatic insects may play a role in transmitting BU to humans (12,13). Naturally acquired *M. ulcerans* infection in wild animals (14) suggests that the etiologic agent is an environmental organism. Most authorities divide BU lesions in the skin into three clinical categories: nonulcerative forms (papules, nodules, indurated plaques, or edema), ulcerative forms, and the healing or scarring form (1,6). Bone lesions also exist (15).

Even though large numbers of patients have been reported, the epidemiology of BU remains obscure, even in disease-endemic countries. In 1997, a first report was published on 867 BU patients from the Republic of Benin (West Africa) for 1989–1996 (4). Our study covers the ensuing 5 years (1997 to 2001), during which a collaborative project was initiated to improve detection and control of BU. This study describes BU in Benin and presents demographic trends and epidemiologic data from the four southern regions of Benin (Zou, Oueme, Mono, and Atlantique), as seen in a rural hospital in the Zou Region.

## Patients and Methods

Our observations are based on 1,700 consecutive patients diagnosed with BU and admitted from 1997 to 2001 to the Centre Sanitaire et Nutritionnel Gbemoten (CSNG), at Zagnanado in the Zou Region. Age, sex, origin, date of disease onset as reported by the patient, date of diagnosis, duration of hospitalization, clinical characteristics, and evolution of the disease were recorded. Clinical criteria for suspecting BU included: presence of a chronically developing lesion (several weeks or months), i.e., a "wound that will not heal"; no fever or regional lymphadenopathy; typical nodular, indurated plaque or edematous lesion; one or more painless chronic ulcers with undermined edges or a depressed scar; swelling over a painful joint, which suggested bone involvement; and patient age <15 years; patient living or traveling in a disease-endemic zone.

Seventy patients were excluded from the study: 13 were confirmed to have another disease (5 cases of cutaneous tuberculosis, 4 *M. chelonae* abscesses, 2 cases of mucormycosis, 1 case of cutaneous diphtheria, and 1 osteosarcoma), and 57 had recurrent BU (they constitute a particular group of patients with long hospitalization times or many recurrences), which left 1,630 patients for analysis. We define a recurrent case as occurring in a patient with a previous history of BU who has another lesion at the same or different site of the body within 1 year of completing treatment (16). We define mixed forms as the simultaneous presence of different forms in the same patient at one or multiple body sites.

Specimens of tissue and exudates from 906 patients were analyzed by one or more of the following examinations to confirm the clinical diagnosis: direct smear examination for acid-fast bacilli (AFB), culture, IS*2404* polymerase chain reaction (PCR), and histopathologic examination (17). The remaining 724 cases were diagnosed clinically; all were typical of BU and did not present reasonable differential diagnostic problems.

Demographic data of the general population were taken from the most recent national census (1992) (18). Additional statistical information came from the "Benin Demographic and Health Survey" (19). For the 5-year period 1997–2001, demographic data were derived from 1992 statistics that assumed an annual 3.2% growth rate, corrected by projections of 1996 of the National Institute of Statistics and Economic Analysis of Benin.

Data were analyzed with EpiInfo (Centers for Disease Control and Prevention, Atlanta, GA) and SPSS v. 9.0 (SPSS, Chicago, IL) for Windows. Contingency tables were analyzed by the Pearson chi-square test, and nonparametric tests of Mann-Whitney and Kruskal-Wallis were applied to compare medians of asymmetric distributions. These medians are presented with the first quartile (q1) and the third quartile (q3). Cases were excluded from each analysis when information was missing for a specific variable.

## Results

### Geographic Origin of Patients and Changes in Buruli Ulcer Admissions

**Figure 1:**
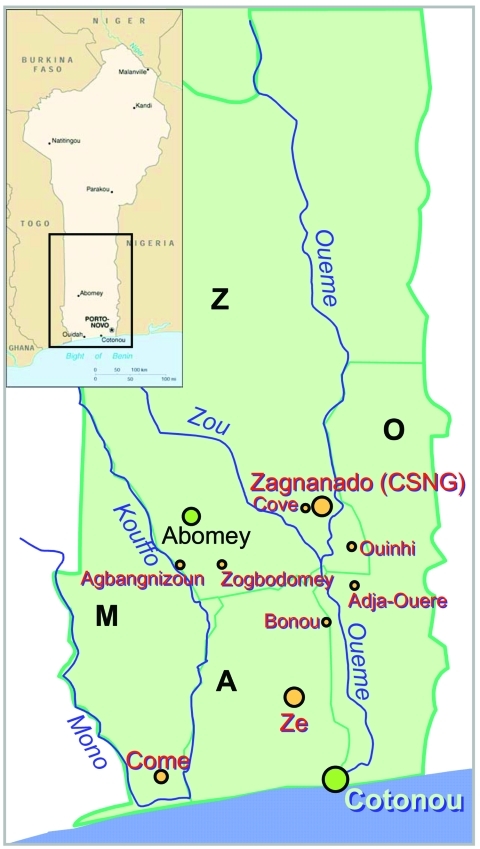
Map of Benin with the four Buruli ulcer–endemic regions: the region of Zou (Z), the region of Atlantique (A), the region of Mono (M), and the region of Oueme (O).

CSNG ordinarily receives patients from the regions of Zou, Oueme, Mono, and Atlantique (Figure 1). Most of the patients whose data were analyzed came from the region of Zou, where CSNG is located, followed by the regions of Oueme, Atlantique, and Mono. Twelve patients were from neighboring countries (Nigeria, Togo, Côte d'Ivoire, and Ghana), and the origin of 24 was not recorded (Table 1). BU has not been reported from the two northern regions of Benin (Atacora and Borgou).

**Table 1:** Origin of Buruli ulcer cases, 1997–2001^a^

Region	1997	1998	1999	2000	2001	Total (%)
Zou	224	229	221	196	195	1,065 (66.3)
Oueme	35	45	90	46	64	280 (17.4)
Atlantique	11	26	35	25	55	152 (9.5)
Mono	17	42	26	6	6	97 (6.0)
Other	3	2	2	3	2	12 (0.8)
Total	290	344	374	276	322	1,606

^a^Of cases in which region of origin was known. A total of 24 cases in the 5-year period were unknown.

**Figure 2:**
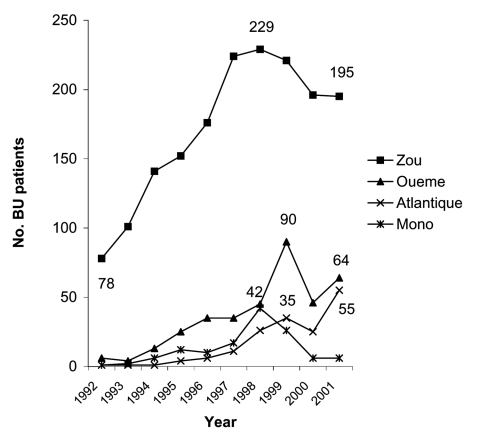
Number of Buruli ulcer (BU) patients by region, 1992–2001.

Figure 2 includes additional data going back to 1992 (4) that show an increased number of patients in all regions of southern Benin from 1992 to 1997, with a decrease in certain regions from 1998 to 2001. A gradual increase is seen in the number of patients from the Oueme and Atlantique regions admitted to CSNG from 1992 through 2001. However, the number of patients from the Zou and Mono regions increased from 1992 to 1998 and then decreased in each region from 1999 to 2001.

Table 2 shows the number of BU patients for some districts of Oueme, Atlantique and Zou regions. For the Zou region, detection rates are also presented. In this region, the number of BU patients coming from Abomey district remained relatively constant from 1997 to 2001. The number of BU patients from Zogbodomey and Agbangnizoun increased. During the same period, the number of patients from Zagnanado and Ouinhi decreased. In the Oueme region, patients from Bonou district increased, and patients from the Adja-Ouere district decreased. In the Atlantique region, the number of patients coming from the Ze district increased notably from 1997 to 2001.

**Table 2:** Changes in number of Buruli ulcer patients in some districts of the Zou, Oueme, and Atlantique regions, 1997–2001^a^

Region	District	1997, N (DR)	1998, N (DR)	1999, N (DR)	2000, N (DR)	2001, N (DR)
Zou	Abomey	11 (13.9)	12 (14.7)	16 (19.0)	10 (11.5)	13 (14.6)
	Agbangnizoun	15 (26.2)	11 (18.6)	22 (36.2)	16 (25.5)	22 (34.1)
	Ouinhi	73 (204.3)	65 (176.6)	53 (139.8)	38 (97.3)	32 (79.6)
	Zagananado	76 (185.9)	68 (161.5)	37 (85.3)	41 (91.8)	52 (113.0)
	Zogbodomey	18 (25.8)	23 (32.0)	32 (43.2)	32 (42.0)	25 (31.8)
Oueme	Adja-Ouere	7	14	15	6	4
	Bonou	23	23	58	29	32
Atlantique	Ze	3	14	22	21	47

^a^DR, detection rate per 100,000 population.

**Figure 3:**
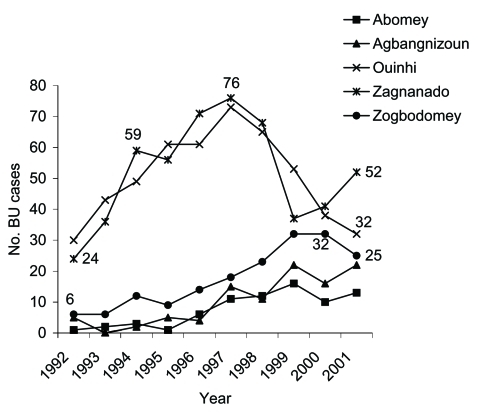
Number of Buruli ulcer (BU) patients from five districts of the Zou Region who were admitted to the Centre Sanitaire et Nutritionnel Gbemoten (Benin), 1992–2001.

**Figure 4:**
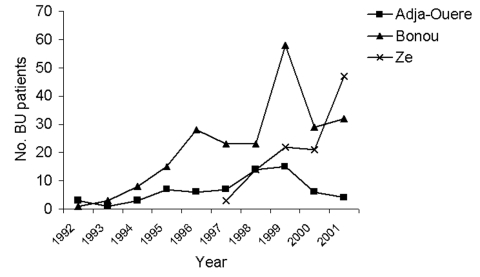
Buruli ulcer (BU) in two districts of the Oueme region and one district of Atlantique region, 1992–2001.

Data from 1992 are represented in Figure 3 for five districts in the Zou region and in Figure 4 for two districts in the Oueme region and one district in the Atlantique region. In the five districts in Zou, the number of patients coming to CSNG was higher in 1997 than in 2000, except for Zogbodomey, where the number increased. From 1992 to 1997, the number of patients coming from Zagnanado and Ouinhi districts increased. After 1997, these numbers decreased to the 1992 level. For the three other districts, the number of BU patients progressively increased from 1992 to 2001. Numbers of BU patients were highest in 1999 in the two districts of Oueme. The number of patients coming from Ze in the Atlantique region doubled between 2000 and 2001. Data before 1992 are not reported because they concern only 71 patients (4).

### Clinical Form of Buruli Ulcer

Different forms of the disease are presented in Table 3. Over the observation period, the percentage of ulcers decreased from 41.0% to 18.3% while mixed forms increased from 12.4% to 24.8%. The percentages of nodules decreased from 1997 to 2001. A total of 7.6% of the patients had osteomyelitis with no active cutaneous form. The percentage of patients with osteomyelitis reached 13.2% when all patients were included. The clinical form of the lesions was not reported for 19 patients.

**Table 3:** Clinical signs and symptoms of Buruli ulcer by year

Clinical form	1997, n (%)	1998, n (%)	1999, n (%)	2000, n (%)	2001, n (%)	Total, n (%)
Nodule	35 (12.1)	35 (10.1)	13 (3.5)	20 (7.2)	19 (5.9)	122 (7.6)
Edema	1 (0.3)	6 (1.7)	1 (0.3)	3 (1.1)	1 (0.3)	12 (0.7)
Plaque	66 (22.8)	98 (28.2)	123 (32.9)	72 (25.9)	111 (34.4)	470 (29.1)
Ulcer	119 (41.0)	99 (28.4)	109 (29.1)	72 (25.9)	59 (18.3)	458 (28.4)
Bone	29 (10.0)	23 (6.6)	31 (8.3)	16 (5.8)	23 (7.1)	122 (7.6)
Mixed	36 (12.4)	69 (19.8)	78 (20.9)	79 (28.4)	80 (24.8)	342 (21.2)
Healed ulcer	4 (1.4)	14 (4.0)	14 (3.7)	15 (5.4)	29 (9.0)	76 (4.7)
Other	0	4 (1.1)	4 (1.1)	1 (0.4)	0	9 (0.6)
Total bone^a^	40 (13.8)	49 (14.1)	55 (14.7)	35 (12.6)	34 (10.5)	213 (13.2)
Total	290	348	373	278	322	1611

^a^Combined osteomyelitis with no associated active cutaneous form and mixed forms with bone lesions.

If bone and mixed forms are divided into ulcerated and nonulcerated forms, the percentages of ulcerated and nonulcerated forms remained relatively constant for the entire study period. No statistical difference was found between the percentage of ulcerated and nonulcerated forms from 1997 to 2001 (data not shown).

**Figure 5:**
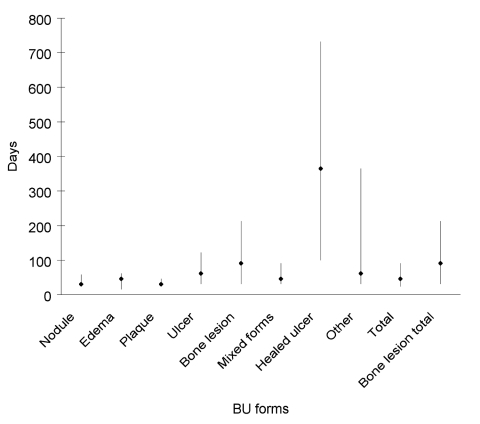
Median patient delay and interquartile range by Buruli ulcer (BU) clinical form.

Delay in seeking medical attention was related to clinical form of the disease (Figure 5) (all patients during entire study period). Median was 30 (q1 = 23, q3 = 58) to 46 days (q1 = 15, q3 = 101) for nonulcerated forms (nodule, edema, and plaque) and 61 days (q1 = 30, q3 = 122) for ulcerated forms. Median delay for bone lesions was 91 days (q1 = 30, q3 = 213).

**Figure 6:**
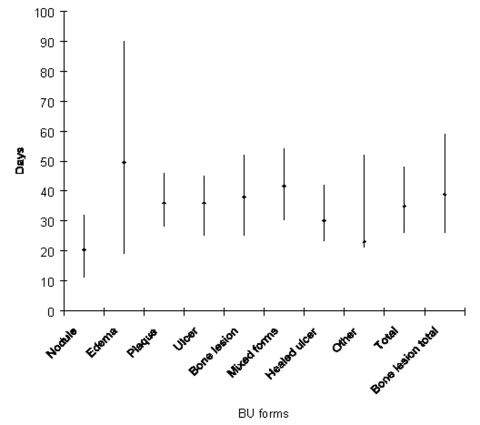
Median duration of hospitalization and interquartile range by Buruli ulcer (BU) clinical form.

Comparison of the duration of hospitalization with the clinical form is shown in Figure 6. Except for patients with a nodule, who spent 20.5 days (q1 = 11, q3 = 32) at the hospital, median times of hospitalization for all patients with each form of disease during the study period was from 23.0 (q1 = 21, q3 = 52) to 49.5 days (q1 = 18.5, q3 = 90).

**Figure 7:**
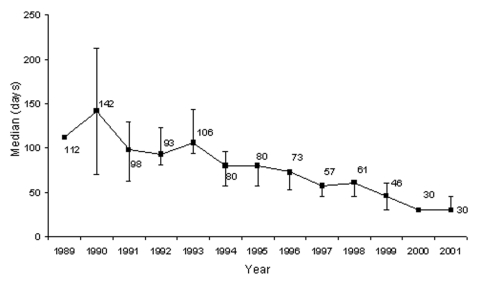
Median patient delay, Centre Sanitaire et Nutritionnel Gbemoten (Benin), 1989–2001.

**Figure 8:**
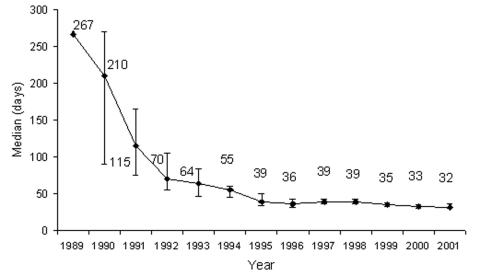
Median duration of hospitalization for Buruli ulcer, Centre Sanitaire et Nutritionnel Gbemoten (Benin), 1989–2001.

Median patient delay in seeking medical care (for all clinical forms) over the study period was 46 days (Table 4). In 1997, median delay was 57 days, while in 2001 delay was reduced to 30 days. Overall median delay at CSNG from 1989 to 2001 declined from approximately 4 months to 1 month (Figure 7). Median hospital stay at CSNG from 1989 to 2001 declined from approximately 9 months to 1 month (Figure 8).

**Table 4:** Changes in patient delay and duration of hospitalization in Buruli ulcer patients, 1997–2001

Year	Median patient delay (q1–q3)	p	Median duration of hospitalization (q1–q3)	p
1997	57 (30–91)		39 (31–53)	
		NS^a^		NS^a^
1998	61 (30–91)		39 (28–54)	
		NS^a^		< 0.001^a^
1999	46 (23–122)		35 (26–43)	
		0.001^a^		0.009^a^
2000	30 (23–61)		33 (23–42)	
		NS^a^		NS^a^
2001	30 (23–61)		32 (24–44)	
Total	46 (23–91)	<0.001^b^	35 (26–48)	< 0.001^b^

^a^Mann-Whitney nonparametric test. Significance level = 0.0125 (Bonferroni correction for pair comparison = 0.05/4). NS = nonsignificant. ^b^Kruskal-Wallis nonparametric test. Significance level = 0.05.

In 2000, the method of referral of BU cases to CSNG was recorded. Patients previously treated at CSNG recommended treatment at CSNG for 68.3% of the patients; 22.1% were referred by a family member acquainted with CSNG, and 5.9% were referred by village outreach activities of CSNG. Only 3.7% of the patients were referred by a government health center or a health professional.

## Discussion

In 1997, Aguiar et al. (4) reported characteristics of 867 BU patients in southern Benin for 1992 to 1996. Our study supplements their data with an analysis of BU patients seen at the same medical center over the succeeding 5 years.

As was shown in 1992 to 1996, data collected from 1997 to 2001 indicate that CSNG receives patients mainly from the Zou region, where the center is located. Patients choose CSNG for a variety of reasons, including accessibility, financial concerns, and cultural compatibility. However, two new developments somewhat altered the data for the two periods. The first development was that in 1998, a new treatment center for BU was established at Lalo in the Mono region. This development moderately decreased the number of patients coming to CSNG from this region. Approximately 400 BU patients were treated at Lalo from 1998 through 2001. The second development was that from 1999 through 2001, more patients from the Atlantique and Oueme regions came to CSNG because of active public health programs that raised awareness of BU and the availability of treatment at the facility. During this period, these regions had no treatment centers.

Active case finding performed in the Zou region in 2000 did not result in an increase in the number of BU patients coming from this region. CSNG is well known and highly respected by the population, but some patients refuse to go to it, usually for cultural reasons. Aujoulat et al. (20) published a report on the psychosocial aspects of health-seeking behaviors of patients with BU in southern Benin. Their study indicates that some patients are reluctant to seek treatment at any health center. In addition, our own experiences confirm that some BU patients actively avoid detection and would never be included in official reports. These patients, therefore, would not be identified by active or passive detection methods. We conclude that rates for the Zou region are a valid estimate of the incidence of the disease, even if the rates are slightly underestimated. A comparison of detection rates of BU in the Zou region with those of leprosy and tuberculosis in 1999 shows a higher rate of BU (21.5/100,000) than of leprosy (13.4/100,000) and tuberculosis (20.0/100,000) (19). Regional differences in the prevalence of BU exist, and the disease is believed to be severely underreported.

A few BU-endemic countries have reported national data on prevalence and incidence. For Uganda in 1972, Barker (8) reported incidence >500/100,000 in some regions. In Ghana, Amofah et al. (5) estimated a prevalence of 22% in some villages of the Amansie District and a national prevalence of 20.7/100,000 (21). Marston et al. (7) found a local prevalence of 16.3% in the Daloa region of Côte d'Ivoire. Seasonal variations in the frequency of BU have been reported in several countries (9,10). Environmental alterations may cause changes in BU frequency (9). Moreover, search for environmental *M. ulcerans* DNA detected variations in positivity rates of specimens over time, and these changes are reflected in corresponding alterations of frequency of BU patients in the same foci (Portaels et al., unpub. data). The focal nature of BU prevalence is important in determining the overall disease rate.

Discrepancies between some published reports and our data are partly explained by factors that influenced frequencies in different BU-endemic regions in Benin. In the Zou region between 1992 and 1997, treatment facilities at CSNG developed markedly. This effort became even more efficient after 1997, as a result of aid from the Directorate-General for Development Cooperation (DGDC, Belgium), beginning in 1998. In the Mono region, the Médecins Sans Frontières–Luxembourg established a BU treatment facility in 1998 and conducted rural public health training and publicity programs. In the Oueme and Atlantique regions, Raoul Follereau France and Luxembourg Foundations conducted population surveys in 1999 for the future development of treatment centers in these regions.

The drop in frequencies after these peak years, perhaps related to reduced rural public health education activities, fell to pre-1997 levels, when detection was totally passive. This effect could be explained by cyclic environmental changes, such as excessively dry or wet periods that differ from region to region. Data for Ouinhi and Zagnanado in the Zou region have been collected since 1992 (Figure 3). From 1992 through 1996, frequencies in these two districts increased rapidly, probably because more BU patients had become aware of the effective therapy offered by CSNG. Detection of BU reached its highest level in 1997.

Reductions in new cases from Ouinhi and Zagnanado, beginning in 1998, may be attributable to any number of factors. After the intensive publicity on BU was discontinued, inhabitants may have begun to lose interest. After the intensive campaign, traditional practitioners' interest in the disease may have increased, and they may have promoted their treatment methods in their respective villages. Because of fear of surgery and lack of local access to practitioners, patients may initially prefer traditional therapists, who do not perform surgery. Transportation costs are minimized by frequenting local practitioners (22). The active program may have reduced the reservoir of untreated patients. Possibly, disease-endemic sites may have become less contaminated with *M. ulcerans*. Environmental studies show that some disease-endemic sites in the district of Ouinhi became less frequently positive for *M. ulcerans* DNA (Portaels et al., unpub. data).

As shown in Figure 3, BU frequencies remain nearly constant for three districts (Abomey, Agbangnizoun, and Zogbodomey). Frequency in the Ze district (Figure 4) increased in 2001, which may be attributed to active public health publicity campaigns. Abomey and Agbangnizoun districts are in the Kouffo River basin (Figure 1) rather than the Zou and Oueme basins, and Zogbodomey is more closely related to the Kouffo than to the Zou basin. This finding suggests that changes in the BU rates in the Abomey, Agbangnizoun, and Zogbodomey districts remained stable because of common hydrologic relationships. Differences in number of cases coming from the districts of Ouinhi/Zagnanado and Agbangnizoun/Abomey/Zogbodomey could be related to uninvestigated environmental differences in the two different basins.

Other scientists (23,24) reported osteomyelitis in BU patients; in this study, bone involvement was frequent (13.2%). As shown in Figure 5, the form of disease is related to the period of delay in seeking medical attention. Nonulcerated forms have a median delay of 1 to 1.5 months, ulcers 2 months, and patients with osteomyelitis 3 months. This finding has several possible explanations. The nonulcerated form is the first stage of the disease in the nodular, edematous, or plaque form. After a variable period of time (a few weeks to several months), these forms ulcerate. Also, disseminated bone lesions take approximately 3 months to develop. Because open skin lesions may not be visible at the site of the bone lesion, the disease may go undetected or disregarded for long periods. Lesions may also arise by reactivation of subclinical latent foci (15).

In 1997, Aguiar et al. (4) described 867 BU cases, of which 94% were ulcerated. Improved knowledge on clinical classification of BU has led to recognizing a higher percentage of nonulcerated and mixed forms and fewer patients with ulcers. Nodules are less common in Benin. The present study shows that the percentage of ulcerated and nonulcerated forms of the disease was approximately 50% from 1997 to 2001 and that the fluctuation in the percentage of ulcerated and nonulcerated stages was insignificant. We attribute the difference in the percentage of ulcerated forms before 1997 to delayed admission to the hospital in 1989 to 1996. However, in spite of the reduced delay in admission after 1997, we have not observed an increased number of nodules. The reason for the reduced rate of nodular disease in Benin remains obscure.

Median patient delay in admission to hospital decreased from 1997 through 2001. From 1998 through 1999, the difference was not significant, but it became significant between 1999 and 2000 (Table 4). Introduction of the DGDC's "Ulcère de Buruli au Bénin" Program in 1998 was an important factor in the marked reductions in patient delay. Moreover, in 2000, promotional sessions on BU were organized by DGDC and the National BU Program PNLUB (Programme National de Lutte contre l'UB) in the Zou, Oueme, and Atlantique regions. After these efforts, patients reported earlier to the center than in 1999.

Median duration of hospitalization decreased from 1997 through 2001. These changes could be attributed to reduced patient delay (earlier care-seeking by patients with less severe lesions, especially those with ulcers and bone involvement) or improved patient care at the health center. Except for patients with nodules, patients with all other forms of the disease are usually hospitalized for 1 to 2 months (Figure 6). Under field conditions in BU-endemic countries, we believe this period of hospitalization is unlikely to be further reduced significantly for advanced BU disease with the current therapies.

Referral of patients to CSNG for treatment depended largely on word-of-mouth suggestions by former BU patients. Patients now tend to bypass traditional therapists and go to CSNG. Accepting surgical treatment remains a deterrent to seeking institutional therapy (20). In these cases, former patients often provide the incentive to seek appropriate therapy. In 2000, a high percentage (68.3%) of patients was referred to CSNG by a previously treated patient.

While some health workers suggest that clinical features are sufficient to diagnose BU, in our experience, bacteriologic and histopathologic evaluations remain important for disease confirmation. This fact is especially true for all research projects on BU. Numerous conditions may present differential diagnostic problems, including parasitic infections, mycotic diseases, neoplastic conditions, tropical phagedenic and stasis ulcers, and cutaneous tuberculosis. In our study a disease other than BU was confirmed in 13 (1.4%) of 906 patients by bacteriologic or histopathologic analysis.

In conclusion, data from a rural hospital at Zagnanado show that BU is highly endemic in southern Benin. Our study highlights the importance of a team approach for optimal management of *M. ulcerans* disease, both at the village and treatment center levels. Such strategies should include efforts in early diagnosis and effective therapy that are compatible with the socioeconomic structure (25). These goals were largely achieved in Benin because of the implementation of an International Cooperation Program and the creation of a national BU Program.

We believe that a multidisciplinary approach that involves educating the population, training healthcare workers, adequately managing cases, and simplifying surgical procedures reduced hospitalization time and stimulated patient initiative. All these approaches improve patient outcome and lower the socioeconomic effect of the disease on rural populations.
